# RNA-Sequencing of Primary Retinoblastoma Tumors Provides New Insights and Challenges Into Tumor Development

**DOI:** 10.3389/fgene.2018.00170

**Published:** 2018-05-17

**Authors:** Sailaja V. Elchuri, Swetha Rajasekaran, Wayne O. Miles

**Affiliations:** ^1^Department of Nanotechnology, Vision Research Foundation, Sankara Nethralaya, Chennai, India; ^2^The Ohio State University Comprehensive Cancer Center, Columbus, OH, United States; ^3^Center for RNA Biology, The Ohio State University, Columbus, OH, United States; ^4^Department of Molecular Genetics, The Ohio State University, Columbus, OH, United States

**Keywords:** Retinoblastoma, RNA-sequencing, non-coding RNA (LINC-RNA), tumor, RB1, metabolic profiling

## Abstract

Retinoblastoma is rare tumor of the retina caused by the homozygous loss of the Retinoblastoma 1 tumor suppressor gene (RB1). Loss of the RB1 protein, pRB, results in de-regulated activity of the E2F transcription factors, chromatin changes and developmental defects leading to tumor development. Extensive microarray profiles of these tumors have enabled the identification of genes sensitive to pRB disruption, however, this technology has a number of limitations in the RNA profiles that they generate. The advent of RNA-sequencing has enabled the global profiling of all of the RNA within the cell including both coding and non-coding features and the detection of aberrant RNA processing events. In this perspective, we focus on discussing how RNA-sequencing of rare Retinoblastoma tumors will build on existing data and open up new area’s to improve our understanding of the biology of these tumors. In particular, we discuss how the RB-research field may be to use this data to determine how RB1 loss results in the expression of; non-coding RNAs, causes aberrant RNA processing events and how a deeper analysis of metabolic RNA changes can be utilized to model tumor specific shifts in metabolism. Each section discusses new opportunities and challenges associated with these types of analyses and aims to provide an honest assessment of how understanding these different processes may contribute to the treatment of Retinoblastoma.

## Introduction

Tight control over proliferation and differentiation processes are fundamentally important to modulating organismal development and prevent oncogenic growth. In eukaryotes, the Retinoblastoma 1 (*Rb1*: gene, pRB: protein) gene and the E2 promoter binding Factors (E2Fs) function to control both of these pathways (Hiebert et al., 1992; Du et al., 1996). The pRB protein is the sole protein which can bind to and repress the activity of the activator E2Fs, E2F1-E2F3 (Hiebert et al., 1992), and this has made inactivation of the pRB pathway in cancer almost ubiquitous. Loss of pRB function, permits the constitute activity of the activator E2Fs and the uncontrolled proliferation of cells. Additionally, pRB-deficient cells have developmental defects which commonly prevents these cells from terminally differentiating (Xu et al., 2009, 2014). pRB binding to the activator E2F’s mediates the recruitment of numerous transcriptional repressor complexes including histone deacetylases (HDAC) and mating-type switching (SWI) and Sucrose Non Fermenting (SNF) (SWI/SNF) promoting repressive chromatin modifications on E2F target genes (Brehm et al., 1998). In cancer cells the pRB pathway is disabled by numerous mechanisms including; inactivating and/or gene mutations with in the Rb1 loci (Friend et al., 1986), the E7 oncoprotein produced by the Human Papilloma Virus (Dyson et al., 1989), the amplification or overexpression Cyclin D and/or Cyclin Dependent Kinase 4 or 6 (CDK4 and CDK6) (Nobori et al., 1994; Connell-Crowley et al., 1997; Harbour et al., 1999) or by the deletion or silencing of CDK inhibitors, CDKN2A-D (Cairns et al., 1994). Inactivation of the pRB/E2F pathway is one of the hallmarks of cancer and is ubiquitously disrupted in most tumor types (Sherr, 2000; Classon and Harlow, 2002). New research focusing on how pRB-loss changes cells has identified novel pathways sensitive to pRB-function including genome stability (Manning et al., 2010), metabolism and mitochondria biogenesis (Nicolay et al., 2015; Varaljai et al., 2015). The characterization of these unexpected biological processes coupled with the identification of new pRB interacting partners and the expansion of transcriptional profiling quality have inspired the pRB-field to probe how pRB-function modulates every aspect of cellular biology.

The RB1 gene was originally cloned as the genetic cause of the pediatric tumor of the retina, Retinoblastoma (Friend et al., 1986; Lee et al., 1987). Homozygote loss of the RB1 gene within these cells cause their aberrant proliferation and incorrect specification their cell fate (Xu et al., 2009, 2014). A small subset (3%) of Retinoblastoma tumors retain pRB function and contain MYCN amplifications (Bowles et al., 2007). RB1-deficient Retinoblastoma tumors are found in both heritable and sporadic forms (Friend et al., 1986; Lee et al., 1987) and occurs at the incidence of 1 in 15,000–20,000 births (Dimaras et al., 2012). These patients are also frequently affected by secondary tumors during adolescence that are commonly sarcoma in origin but can also affect a number of organs including the lung, bladder, and brain (Eng et al., 1993; Nahum et al., 2001). These secondary tumors are frequently metastatic and these patients have poor long term prognosis.

Genomic studies and microarray data have identified rod and cone retinal pigment epithelial (RPE) cells as the major cells of origin of Retinoblastoma (Xu et al., 2009, 2014; Kapatai et al., 2013; Cobrinik, 2015). High resolution Single nucleotide polymorphism (SNP) arrays from both hereditary and non-hereditary tumors show large genetic variation between these groups and suggest sub-categorization is possible (Kooi et al., 2015). In particular, hereditary Retinoblastoma tumors have less genomic rearrangements whilst non-hereditary tumors exhibit higher levels of genomic instability (Corson and Gallie, 2007; Mol et al., 2014). These genomic changes predominantly result in the gain of a number of chromosomal regions including, 1q, 2p, 6p, and 13q and the loss of 16q region (Kooi et al., 2016). Multiple studies have investigated these genomic aberrations and several candidate genes have been identified within these loci including KIF14, MDM4, MYCN, E2F3, DEK, and CDH11. Additional whole genome sampling arrays (WGSA) have found regions of genomic change in Retinoblastoma tumors and identified genes involved in differentiation regulation such as CEP170, SIX1, and SIX4 (Ganguly et al., 2009). This analysis lead to new genomic and epigenetic mapping experiments and to the identification of Spleen tyrosine kinase (SYK) as novel therapeutic target (Zhang et al., 2012; Pritchard et al., 2014). Additional studies linked the Mouse double minute 2 homolog (MDM2) and Tumor Protein 53 (p53) signaling node as being dysregulated in Retinoblastoma’s (de Oliveira Reis et al., 2012; Qi and Cobrinik, 2017) and therapeutic intervention by targeting this pathway resulted in apoptosis of RB cells. These technologies have enable the identification of new and potential therapeutically targetable pathways in Retinoblastoma (Pritchard et al., 2014; Assayag et al., 2016) (reviewed by (Theriault et al., 2014) however, a number of addition key area’s remained unexplored. These include: how do non-coding RNAs changes in these tumors? Can metabolic gene expression be used to predict targetable metabolic defects? And how do RNA processing events change in tumors lacking the RB1 gene? In this perspective, we will discuss the importance of addressing these areas and then highlight some of the challenges that accompany them. Information is power and this is particularly important when trying to improve the clinical outcomes for patients with pediatric tumors.

## RNA-Sequencing Technology

**FIGURE 1:**
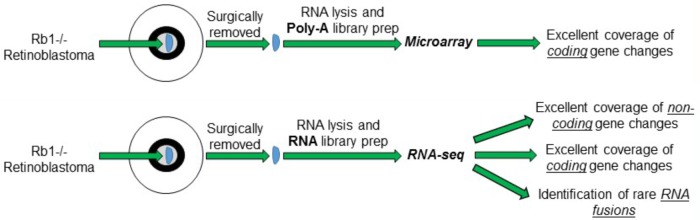
Schematic of the different RNA measurements from Retinoblastoma tumors using conventional microarrays vs. RNA-sequencing.

Pioneering studies into the transcriptome of Retinoblastoma tumors using microarrays have provided critical foundations for our understanding of the disease. The advent of new and unbiased profiling of the RNA content of cells by RNA-sequencing (RNA-seq) significantly increases the resolution of the RNA measurements and can be expanded to include non-coding expression and detection of rare RNA fusion products. RNA-seq provides enhanced transcriptomic profiling by directly sequencing the RNA or DNA copies of the RNA and comparing it to the genome rather than relying on probe detection on traditional microarrays. This technology therefore circumvents the need to test RNA on pre-determined gene sets and can interrogate the entire transcriptome. RNA-seq platforms can measure all the RNA of cells, however, require the removal of very abundant RNA species within the cells that do not contribute to the transcriptome including ribosomal RNA (rRNA) and transfer RNA (tRNA). This critical experimental step depletes these specific RNA species from the cellular pool of RNA before library preparation and increases the overall coverage and depth of RNA-seq reads on the remaining genes. Currently RNA-seq datasets of Retinoblastoma tumors and normal controls have not been published, however, several independent teams are known to be working to generate these exciting datasets. In this perspective, we discuss three exciting opportunities that RNA-seq datasets of Retinoblastoma tumor may provide to the research community (**Figure 1**).

## Long Intragenic Non-Coding RNA Changes

A number of leading laboratories around the globe have used microarray technology to determine the transcriptional changes in RB1-/- Retinoblastoma samples compared to normal retinal controls (Ganguly and Shields, 2010; Zhang et al., 2012). This data has been invaluable to understanding how changes in the mRNA composition of these tumors contribute to the underlying biology driving the aberrant proliferation and differentiation of RB1-/- retinal cells. These tools do provide excellent coverage of the coding transcriptome, however, their capacity to detect non-coding RNA changes is limited to a small number of highly abundant long intergenic non-coding RNAs (LINC-RNAs). A growing body of literature has highlighted that changes in the non-coding RNAs including microRNAs (miRNAs) and LINC-RNAs may have important and clinically predictive roles in tumorigenesis. In particular, LINC-RNA integrated maps have enabled researchers to sub-divide Head and Neck Squamous Cell Carcinoma patients into different sub-groups based upon their LNC-RNA profile and this has enabled the identification of LINC-RNAs associated with poor prognosis (de Lena et al., 2017). In Retinoblastoma tumors only a limited number of studies of LINC-RNAs have been possible due to their poor representation on microarrays and this work has focused on LINC-RNAs identified in other tumor types including CCAT (Zhang et al., 2017), BANCR (Su et al., 2015), H19 (Zhang et al., 2018), MALAT1 (Liu et al., 2017), and HOTAIR (Dong et al., 2016). Therefore determining how the spectrum of LINC-RNA expression changes in Retinoblastoma tumors is an area of growing interest. This is particularly important as a subset of Retinoblastoma patients will develop life threatening sarcomas of the muscle, connective or bone tissue during early adulthood and examining new approaches to identify these patients may be of clinical benefit.

### RB1-Loss and LINC Expression

Homozygote inactivation of the Rb1 gene is the initial event triggering the development of Retinoblastoma. As discussed in the introduction, pRB acts as a transcriptional repressor to inhibit the function of the activator E2F transcription factors (E2F1-3). E2F1-3 are potent inducers of transcription and in particular genes required for cell cycle progression and apoptosis, however, very little is known about how these factors control LINC-RNA expression. Preliminary studies from cell lines mapping activator E2F-mediated regulation of LINCs have suggested that a significant number of these RNAs may show transcriptional changes upon RB-loss via both direct and in-direct mechanisms (Feldstein et al., 2013; Bida et al., 2015; Gasri-Plotnitsky et al., 2017). In addition, genome-wide E2F Chromatin Immunoprecipitation (ChIP) experiments have identified both activator and repressor E2F binding in genomic loci that contain LINC-RNAs (Xu et al., 2007). These preliminary studies have provided tantalizing hints that E2F and RB modulation of LINC-RNAs is widespread and potentially important for cells. Clearly, much more work needs to be done to determine how the entire family of E2F transcription factors (both the activators and repressors) and the RB-like proteins (RBL1 and RBL2) function to modulate the levels of non-coding RNAs both during developmental and oncogenic processes.

### LINC Expression in Retinoblastoma Tumors

Given that our understanding of how RB1 loss *in vitro* or in model organisms changes the LINC expression profile is limited, determining how RB1 homozygote mutation affects LINC levels in a complex and developmentally disorganized Retinoblastoma tumor represents a real challenge. With the development of RNA-sequencing that unbiasedly measures the levels of RNA in cells without the need to purify polyadenylated mRNAs or have probes designed against the transcriptome we can now, for the first time, interrogate the entire transcriptome of these tumors. This new approach will enable the development of Retinoblastoma specific LINC-RNA profiles and may generate clinically predictive biomarkers for identifying patients likely to develop secondary tumors. However, significant technical challenges have arisen in other tumor types when profiling LINC-RNAs. These are generally due to the lower expression levels of most LINC-RNAs compared to coding mRNAs and the high variability between patients. RNA-seq reads at the lower end of the runs tend to have greater signal to noise levels than better expressed mRNAs which can make generating predictive LINC profiles from small numbers of tumors difficult. This is a potential issue in Retinoblastoma studies as these tumors are rare and both normal control and tumor cohort size tends to be smaller than for more common adult malignancies.

**FIGURE 2:**
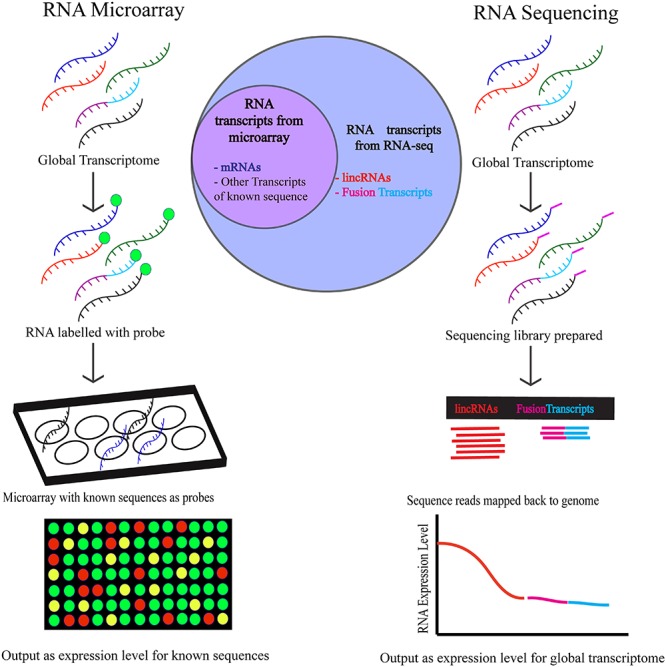
The workflow of RNA-microarray and RNA-sequencing is depicted above. Higher number of targets are detected by RNA-sequencing using which targets of unknown sequence and low abundance targets can both be identified. To detect targets using RNA microarrays, the sequence of the transcripts must be known and expressed in abundance in the cell.

Although there are currently significant gaps in our understanding of how RB1 and the E2F pathway modulate the expression of the LINC-RNA transcriptome in cells and in tumors there seems to be little doubt that expanding our knowledge about how RB1-loss changes the entire transcriptome is an exciting and clinically relevant question (**Figure 2**). Profiling Retinoblastoma by RNA-seq technology will help fill some of the gaps in our knowledge and provide new insights into how RB1-loss may change the LINC-RNA transcriptome of these aggressive tumors but also to those in more genetically complex adult tumors.

## Gene or RNA Fusions

In addition to the documentation of LINC-RNA and other non-coding RNA changes caused by RB1 loss, RNA-seq also enables the measurement and identification of novel or fusion RNAs in samples. The pRB protein has important roles in modulating genomic integrity and stability and loss of pRB has been shown to contribute to accelerated genome manipulations and enhanced drug sensitivity. RNA-seq of Retinoblastoma tumors may also enable the identification of novel RNA species caused by genomic rearrangements or aberrant RNA processing events (**Figure 2**). Such events would normally be excluded or undetectable with traditional microarray technology. Finding and mapping these rare events is an area of growing interest as the aberrant proteins are potential neo-antigens which can be recognized by the patients’ immune system. A number of groups have highlighted the utility of these “abnormal” products for the clearance of tumors, although as yet not in Retinoblastoma, to immunotherapy. This is an emerging clinical avenue and in particular could have significant advantages in the treatment of Retinoblastoma as it would reduce the exposure of pediatric patients to developmentally harmful chemotoxic treatments. The detection and mapping of these events is possible with deep RNA-seq coverage and may provide insights into how splicing and/or RNA metabolism is changed in Retinoblastoma tumors.

## Alternate Splicing Events in Retinoblastoma

Global profiles of splicing changes in Retinoblastoma tumors have yet to be published. Alternate splicing events in the Retinoblastoma cell line, Y79, using vector capping methodology revealed several variants in 57 Eye related genes including transcriptional factors, signal transduction proteins, membrane and secretory proteins (Oshikawa et al., 2011). Several discrepancies were observed in transcriptional start sites of some of these genes and splice variants were produced due a number of aberrant splicing events including lack of exons, insertion of exons, shifting of splice sites and non-splicing events. Additional studies from the same cell line found that an alternatively spliced form of Disabled-1 (Dab1) changed the phosphorylation levels of the Src family of kinases resulting in aberrant signaling (Katyal et al., 2011). When these Y79 cells were differentiated into neuronal cells, different splice variants of Ca(v)3.1 were observed indicating tissue specific alterations in Ca(2+) signaling (Bertolesi et al., 2006). Despite these studies, much more work is required to determine the global mRNA alternative splice variants in Retinoblastoma (McEvoy et al., 2012; Rodriguez-Martin et al., 2016; Cygan et al., 2017). Therefore, there is a significant need for studying these events using latest RNA Seq methods in Retinoblastoma tumors.

## Metabolic Pathway Prediction

The in-depth profiles of transcriptional changes in Retinoblastoma tumors would be a significant resource to the community. In this section, we detail how these datasets could be utilized to make testable predictions into the molecular reprograming of Retinoblastoma cells. Microarray transcriptional profiling and proteomics analysis of Retinoblastoma tumors have identified a number of putative metabolic changes within the tumor cells (Kooi et al., 2015; Danda et al., 2016). From these studies, dysregulated lipid metabolism, mitochondrial energy metabolism and photoreceptor metabolism were implicated as key processes linked to Retinoblastoma progression. These findings are supported by similar findings in RB1 mutant animal models and cell lines (Nicolay et al., 2015) that identified changes in lipids, Glycolysis, amino acid and TCA cycle metabolism (Nicolay and Dyson, 2013; Nicolay et al., 2013, 2015; Kohe et al., 2015; Kohno et al., 2016; Muranaka et al., 2017). Collectively, these results suggest that understanding metabolic pathway changes or reprogramming events in Retinoblastomas may provide new therapeutic opportunities.

Compared to the high throughput methods available for studying global genomic, epigenetic and transcriptional changes in cancer progression metabolomics is an evolving discipline in cancer research. The major challenge for determining metabolic defects in primary tumor samples is the inability to measure metabolic flux using radioactive Carbon or Nitrogen molecules. In an attempt to circumvent these limitations new computational systems biology approaches could be used to understand metabolic reprogramming in Retinoblastoma (Medina-Cleghorn and Nomura, 2013). System biology employs constraint-based modeling that requires reconstruction of the metabolic reactions occurring in a cell followed by computational simulations and experimental validations of the models (Hernandez Patino et al., 2012). Among the models, flux balance analysis gained importance and has been successfully employed to predict metabolic state alteration in cultured cancer cells (Duarte et al., 2007; Orth et al., 2010; Resendis-Antonio et al., 2010; Hu et al., 2013). Using integrated system biology approaches for metabolic modeling of Retinoblastoma tumors may provide new opportunities to develop personalized treatment options for Retinoblastoma patients (Filipp, 2017).

Utilizing the excellent coverage and depth of RNA-seq data from Retinoblastoma tumors and normal tissue, we will be able to generate system level metabolic models, termed genome –scale metabolic models (GEMs). This approach has previously been employed to assay model organisms, tissue and drug treatment network maps (Agren et al., 2014; Bordbar et al., 2014; Mardinoglu et al., 2014; Varemo et al., 2016; Hinder et al., 2017). One limitation of this approach is the assumption that gene expression levels correlate to protein levels (Fagerberg et al., 2014). However, protein abundance variation due to post-transcriptional regulation and/or post-translational modification is not included in this model building. Despite this limitation, RNA-seq data has previously been used to develop Task-driven Integrative Network Inference for Tissues (tINIT) algorithms and draft GEM profiles. Once generated these networks can then be compared with other models from both experimental results and the scientific literature to determine the overall accuracy of the predictions. From this, 65 draft cell type-specific metabolic models consisting of 2,426 ± 467 reactions (± s.d.) and 1,262 ± 204 transcripts have been established (Shlomi et al., 2008; Schellenberger et al., 2011). By constructing these profiles we can begin to build Retinoblastoma specific predictions of the metabolic changes in these tumors that can be experimentally tested. Additionally, several layers and cell types in retinal tissue perform unique functions necessitating the development of a sub-network in cell dependent manner. Research generating a cell specific GEM in retina cells may enable modeling of other ocular diseases including Age related Macular Degeneration, Glaucoma Diabetic retinopathy, Uveal melanoma and Retinoblastoma. RNA-seq analysis of these ocular diseases has identified several unique retinal genes (Farkas et al., 2013; Li et al., 2014). Utilizing existing approaches and developing RNA-seq datasets from Retinoblastoma tumors may allow the development of Retinoblastoma specific disease GEMs that may open avenues for developing new therapeutic opportunities. The predictive tools are very sensitive to biological variations that occur in different cohorts necessitating measurements of metabolites using additional high through techniques. Alternatively, the experimental metabolite data can be integrated into modeled metabolic reaction networks to understand diseased condition. A similar approach led to the generation of subnetwork responsible for impaired glucose tolerance in patients compared to normal condition (Deo et al., 2010). Utilizing high coverage RNA-seq data from Retinoblastoma tumors may enable the development of metabolic pathway predictions in this rare pediatric tumor.

## Conclusion

Microarray profiling of Retinoblastoma tumors has been instrumental in identifying the aberrant pathways driving the growth and invasion of the tumors. These approaches have really built an immense foundation that has enabled the Retinoblastoma research field to test and develop new ways to therapeutically target these tumors. This technology, that has served us so well, has a number of limitations that can be largely circumvented by the use of RNA-sequencing. In particular, RNA-seq measures both coding and non-coding RNAs changes which may be invaluable to understanding the biology of these tumors but also in helping to stratify patients into different treatment outcome or relapse groups. Collectively, this tool enables us to interrogate the biology sustaining Retinoblastoma tumor growth and may led to discovery of new therapeutic approaches to treat these patients.

## Ethics Statement

The protocol was approved by institutional review board on Ethical practices for research at Sankara Nethralaya.

## Author Contributions

SE and WM contributed to the conception, preparation, and revision of the manuscript. SR built the figures. All of the authors approved and agreed on the manuscript.

## Conflict of Interest Statement

The authors declare that the research was conducted in the absence of any commercial or financial relationships that could be construed as a potential conflict of interest.
